# A freaky artery

**DOI:** 10.1007/s12471-018-1188-z

**Published:** 2018-10-22

**Authors:** R. Joustra, A. P. J. van Dijk, H. W. J. Meijburg, M. Boulaksil

**Affiliations:** 1 Department of Cardiology, Radboud University Medical Center, Nijmegen, The Netherlands; 2 Department of Cardiology, Jeroen Bosch Hospital, ’s-Hertogenbosch, The Netherlands

A 77-year-old male patient, with an irrelevant cardiovascular history, was referred to our hospital with acute severe interscapular pain arising at rest. A computed tomography angiography (CTA) was performed (Fig. 1a, b). What is your diagnosis?

**Fig. 1:**
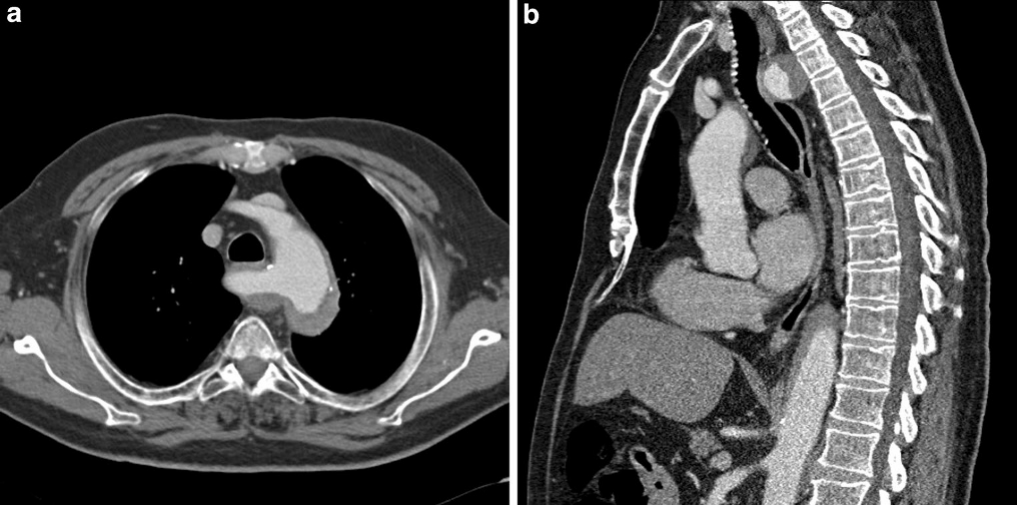
**a** Transversal CT section of our patient. **b** Sagittal CT section

## Answer

You will find the answer elsewhere in this issue.

